# Oral Administration of Lotus-Seed Resistant Starch Protects against Food Allergy

**DOI:** 10.3390/foods12040737

**Published:** 2023-02-08

**Authors:** Jiamiao Hu, Zhongjing Lin, Lanxin Li, Baodong Zheng, Hongliang Zeng, Yanbo Wang, Yi Zhang

**Affiliations:** 1 College of Food Science, Fujian Agriculture and Forestry University, Fuzhou 350002, China; 2 Fujian Provincial Key Laboratory of Quality Science and Processing Technology in Special Starch, Fuzhou 350002, China; 3 School of Food and Health, Beijing Technology and Business University, Beijing 102448, China

**Keywords:** food allergy, lotus seed, resistant starch, Th1/Th2 balance

## Abstract

Food allergy is a serious food safety and public health issue. However, the medical interventions for allergy treatment are still suboptimal. Recently, the gut microbiome–immune axis has been considered as a promising target to reduce the symptoms of food allergy. In this study, we explore the oral administration of lotus-seed resistant starch as a means to protect against food allergy using an ovalbumin (OVA) sensitization and challenge rodent model. The results obtained showed that lotus-seed resistant starch intervention alleviated the food allergy symptoms (such as reductions in body temperature and allergic diarrhea). Furthermore, lotus-seed resistant starch also attenuated the increase in OVA-specific immunoglobulins and improved Th1/Th2 imbalance in OVA-sensitized mice. These anti-allergic effects might be associated with the actions of lotus-seed resistant starch on intestinal microbiota. Taken together, our findings suggest that daily ingestion of lotus-seed resistant starch might be effective for the alleviation of food allergy.

## 1. Introduction

Food allergies comprise a variety of immune-mediated adverse reactions to food, which may be life-threatening in the most extreme cases [1]. Recently, food allergy has become an important public health issue that affects an increasing number of people [2]. Currently, the causes of food allergy are not yet fully understood. However, recent studies have suggested that gut microbial communities play pivotal roles in shaping immune responses against food allergens [3]. For example, Andrew T Stefka et al. identified a common class of gut bacteria, *Clostridia*, which protects against food allergen sensitization by preventing food allergens from entering the bloodstream. Another study also confirmed that *Clostridium butyricum* (a common gut commensal bacterium with the ability to produce butyrate) could reduce anaphylactic symptoms of β-lactoglobulin-induced food allergy in a rodent model by balancing the ratio of Th1/Th2 and Th17/Treg [4]. Similarly, *Lactobacillus rhamnosus* GG could also protect against allergy to cow’s milk by suppressing Th2 response. Indeed, the reshaping of the intestinal bacterial composition by ultra-processed foods and the overuse of antibiotics in modern society are believed to be important factors showing a strong correlation to the sharp rises in food allergy rates in the last several decades. Furthermore, another study also revealed that high dietary fat intake may induce a microbiota signature that promotes food allergy [5].

In this context, scholars have also explored the anti-allergic potential of probiotics and prebiotics. For instance, a commercial lyophilized probiotic mixture consisting of *Lactobacillus gasseri* LK001(40%), *Lactobacillus salivarius* LK002(20%), *Lactobacillus johnsonii* LK003, (15%), *Lactobacillus paracasei* LK004(5%), *Lactobacillus reuteri* LK005(5%), and *Bifidobacterium animalis* LK011(15%) was found to protect against food allergy via the induction of CD103+ mucosal dendritic cells, promotion of regulatory T cell differentiation, and modulation of intestinal microbiota [6]. Meanwhile, a range of functional foods with prebiotic properties (e.g., oligosaccharides [7] and polysaccharides) regulated gut microflora compositions [8].

Resistant starch is a type of dietary fiber that can be fermented by intestinal microorganisms in the large intestine [9]. Indeed, studies have already revealed that the consumption of resistant starches enhances mucosal immunity. In particular, lotus-seed resistant starch has received increasing attention due to its healthy impact on the gut microbiota. For instance, lotus-seed resistant starch was reported to stimulate the growth of beneficial microorganisms (e.g., *Bifidobacterium* and *Lactobacillus*) and reduce the abundance of harmful bacteria in the gut [10]. Moreover, we also found that lotus-seed resistant starch profoundly affects the intestinal short-chain fatty acid production and bile acid pool balance. Considering prior work suggesting that the short-chain fatty acids and bile acids play crucial roles in food allergy regulation, therefore, we hypothesize that lotus-seed resistant starch could be potentially used for the intervention of food allergic reactions by regulating intestinal microbiota.

## 2. Materials and Methods

### 2.1. Drugs and Chemicals

Ovalbumin (OVA, Grade V) was bought from Yuanye Bio-Technology Co., Ltd., Shanghai, China. Aluminum hydroxide (alum) was obtained from Thermo Fisher Scientific, Waltham, MA. All ELISA kits (OVA-specific IgE and IgG1; IL-4, IL-10; TNF-α) were purchased from Wuhan Fine Biotech Co., Ltd., Wuhan, China. The mouse Th1/Th2 staining kit was bought from Multi Sciences Biotech Co., Ltd., Hangzhou, China. The lotus-seed resistant starch was prepared as described in previous studies [11].

### 2.2. Animals

BALB/c mice (6–8 weeks, female) were purchased from Beijing HFK Bioscience Co., Ltd., Beijing, China, and were maintained in the animal center of the College of Food Science, Fujian Agriculture and Forestry University, with stable temperature (23 ± 2 ℃) and humidity (55 ± 10%). Animals were exposed to a 12 h light:12 h dark cycle and allowed ad libitum access to food and water. The mice were allowed to acclimatize to the environment for 1 week before induction of allergic sensitization. All animal experiments were approved and carried out in accordance with the Animal Experimental Ethnics Committee of Fujian Agriculture and Forestry University (registration number: 22-02-28).

### 2.3. Induction of Food Allergy

Mice (5 per group) were sensitized by 2 times intraperitoneal injection of 100 μg OVA mixed with 0.1 mL alum in 0.1 mL PBS on days 0 and 14. Then, the mice were challenged by orally administering 50 mg OVA dissolved in PBS 5 times every two days on days 28 to 40. After OVA exposure, the rectal temperature was measured in 30 min intervals 3 times as indications of the severity of systemic allergic reaction according to a previous method with slight modification [12]. Mice in LRS-treated groups were orally administered with lotus-seed resistant starch (0.3 g/100 g bw) dissolved in distilled water daily from one day after the second sensitization (day 15) until the final oral administration of OVA. Meanwhile, the blank (without induction of food allergy) and the food-allergy-induction groups (with induction of food allergy only) were administered distilled water only.

### 2.4. Evaluation of Food Allergy

The severity of anaphylactic symptoms was estimated by a previously-reported scoring system: 0 = no symptoms, 1 = frequent scratching of ears and nose, 2 = eyes and ears are red and swollen, 3 = red rash or ulceration of mouth and tail, 4 = cramps or muscle contractions, and 5 = shock or death within 30 min. The severity of diarrhea was evaluated according to the scoring system as follows: 0 = normal stool or absent, 1 = slightly wet and soft stool, 2 = unformed stool with moderate perianal staining of the coat, and 3 = watery stool with severe perianal staining of the coat.

### 2.5. Enzyme-Linked Immunosorbent Assay (ELISA)

The serum OVA-specific IgE, IgG1, IL-4, IL-10, and TNF-α levels of mice were determined using enzyme-linked immunosorbent assay method using commercial kits provided by Wuhan Fine Biotech Co., Ltd. (Mouse OVA sIgE kit, Cat No. EM1254 (Indirect ELISA); Mouse OVA sIgG1 kit, Cat No. EM1996 (Capture ELISA); Mouse IL-4 kit, Cat No. EM0119 (Sandwich ELISA); Mouse IL-10 kit, Cat No. EM0100 (Sandwich ELISA); and Mouse TNF-a; kit, Cat No. EM0183 (Sandwich ELISA)) according to the manufacturer’s instructions.

### 2.6. Flow Cytometry

The prevalence of Th1, Th2 cells in spleen cells was detected by flow cytometry using Mouse Th1/Th2 staining kit. In brief, spleen tissues were homogenized in PBS balance solution. The homogenate was incubated in RPMI 1640 cell culture medium containing 10% fetal bovine serum, with 1 µL PMA/Ionomycin Mixture (250×) and 1 µL BFA/Monensin Mixture (250×), mixed, and cultured for 5 h at 37 ℃. Cells were stained by fluorescein isothiocyanate (FITC) anti-CD3, peridinin-chlorophyll-protein complex (PerCP)-cyanine (Cy) 5.5 anti-CD4, R-phycoerythrin (PE) anti-IFN-γ, and Allophycocyanin (APC) anti-IL-4. Measurements were performed using the CytoFLEX LX system, and data were analyzed with CytExpert Software (Beckman Coulter). Cell events were gated using forward-scatter and side-scatter plots. Then, CD3+ CD4+ cells were gated based on FITC and PC5.5 fluorescence intensity before Th1/Th2 cells were gated from the pool of CD3+ CD4+ cells according to IL-4 and IFN-g level (APC and PE fluorescence intensity, respectively).

### 2.7. 16S rDNA Sequencing

The day before the sacrificing of the mice, fecal samples were collected and immediately frozen at −80 ℃. Microbial DNA was extracted from the mice fecal pellets using E.Z.N.A.^®^ bacteria DNA Kit (Omega Bio-tek, Norcross, GA, USA) according to the manufacturer’s protocol. The V3-V4 hypervariable regions of bacteria 16S rRNA gene were amplified with the primers listed in Supplementary Table S1. The obtained amplicon library was prepared with a Sample Preparation Kit (Illumina, San Diego, CA, USA) and sequenced on an Illumina Miseq platform according to standard protocols. Sequences with 97% similarity were defined as an operational taxonomic unit (OTU), and the representative sequence reads of various OTUs were hierarchically classified into different taxa. The data were processed and analyzed on a cloud platform provided by Oebiotech (https://cloud.oebiotech.com/). The obtained 16S data were uploaded to the NCBI database for public access (Accession: PRJNA912321).

### 2.8. Hematoxylin–Eosin Staining

After sacrifice, jejunum tissues were collected and immediately fixed in 4% paraformaldehyde, embedded in paraffin, and cut into slices (5 µm). After dewaxing and rehydration, the slices were stained with hematoxylin–eosin. Images were obtained using an optical microscope at ×100 and ×400 magnification.

### 2.9. Determination of SCFAs and BAs

Fecal SCFAs and bile acid were determined as previously described [13] with necessary modification. Specifically, the SCFAs were extracted from fecal samples using aqueous acetonitrile (50% *v*/*v*) with [2H9]-Pentanoic acid and [2H11]-Hexanoic acid as internal standards. Then, 200 mM 3-nitrophenylhydrazine and 120 mM N-Ethyl-N’- (3-dimethylaminopropyl) carbodiimide hydrochloride-6% pyridine were added before UPLC-MS/MS analysis. The UPLC analysis was performed using the Nexera UHPLC LC-30A UPLC system coupled with ACQUITY UPLC BEH C18 (100*2.1 mm, 1.7 μm) column and AB Sciex Qtrap 5500 tandem mass spectrometer. The mobile phase comprised 0.1% formic acid–aqueous solution (A) and acetonitrile/methanol = 2:1 (B). Chromatographic separation was performed by the gradient elution procedure as given: 0 min A/B (80:20, *v*/*v*), 2 min A/B (80:20, *v*/*v*), 8 min A/B (60:40, *v*/*v*), 8.1 min A/B (5:95, *v*/*v*), 9.5 min A/B (5:95, *v*/*v*), 9.6 min A/B (80:20, *v*/*v*), and 10 min A/B (80:20, *v*/*v*) at 0.4 mL/min. The bile acids were extracted from fecal samples using an aqueous methanol solution (*v*/*v* = 1:1) with CA-d4, 2-Chloro-L-phenylalanine and Lyso PC17:0 as internal standards. The UPLC analysis was also performed using Nexera UHPLC LC-30A UPLC system coupled with Phenomenex Kinetex C18 (2.1 mm × 100 mm, 2.6 µm) column and AB Sciex Qtrap 5500 tandem mass spectrometer. The mobile phase comprised 0.1% formic acid–aqueous solution (A) and methanol: acetonitrile: isopropyl alcohol = 1:1:1 (B) with formic acid (0.1%). Chromatographic separation was performed by the gradient elution procedure as given: 0 min A/B (80:20, *v*/*v*), 0.5 min A/B (80:20, *v*/*v*), 1.5 min A/B (62:38, *v*/*v*), 7.5 min A/B (50:50, *v*/*v*), 10 min A/B (5:95, *v*/*v*), 11 min A/B (5:95, *v*/*v*), 11.01 min A/B (80:20, *v*/*v*), and 12 min A/B (80:20, *v*/*v*) at 0.45 mL/min.

### 2.10. Statistical Analysis

Data were analyzed and presented as means ± standard deviation. Statistical significance of quantitative data was determined using Student’s t-test or one-way analysis of variance (ANOVA) with Tukey’s post hoc, and a *p*-value < 0.05 was considered statistically significant.

## 3. Results

### 3.1. Lotus-Seed Resistant Starch Exerted Anti-Allergic Effects in OVA-Induced Food Allergy Rodent Model

**Figure 1:**
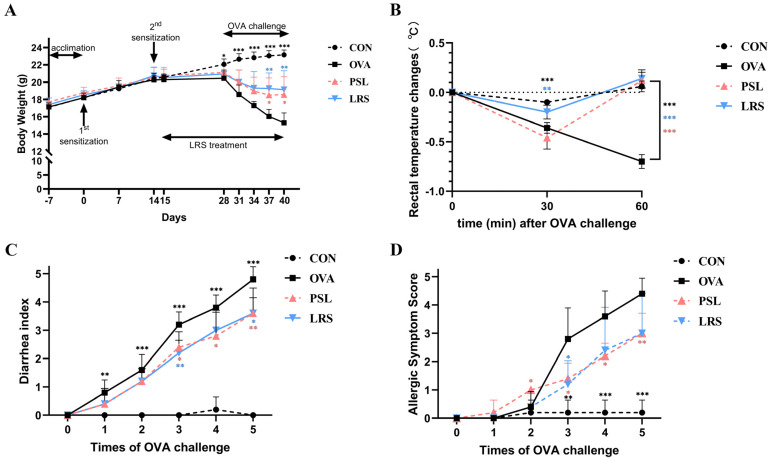
(**A**) Body weight of mice measured over the indicated time course. (**B**) The decrease in rectal temperature induced by ovalbumin (OVA) challenge. (**C**) Diarrhea index of mice following oral OVA challenge. (**D**) Allergic symptom score of mice following oral OVA challenge. CON: control group without ovalbumin sensitization and challenge; OVA: allergic model group with ovalbumin sensitization and challenge; PSL: allergic model mice with prednisolone treatment; LRS: allergic model mice with lotus-seed resistant starch treatment. * *p* < 0.05, ** *p* < 0.01, *** *p* < 0.001 compared to OVA group.

As shown in Figure 1A, all mice showed similar baseline body weight (~17 g) before sensitization. However, significant differences started to appear between non-allergic mice and OVA-sensitized mice from day 28, while the differences remained obvious during the oral challenge (Figure 1A). However, during the induction phase, significant differences were not found between OVA-induced allergic mice with or without lotus-seed resistant starch treatment with respect to the body weight, indicating that lotus-seed resistant starch may have little effect on the body weight of allergic mice.

Meanwhile, exposure to the allergen (OVA) also resulted in decreased rectal temperature (Figure 1B) and severe diarrhea (Figure 1C), as expected. Notably, the rectal temperature decreased upon OVA treatment recovered in 1 h in the mice with lotus-seed resistant starch treatment (Figure 1B). Similarly, the mice with lotus-seed resistant starch intervention also showed a trend of protective effect against diarrhea (Figure 1C) and significantly lower anaphylaxis score than the OVA group (Figure 1D).

**Figure 2:**
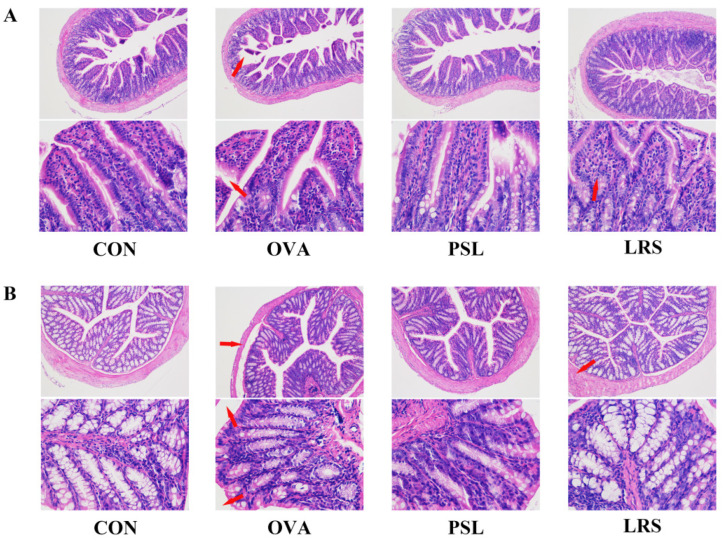
(**A**) Hematoxylin-eosin staining of jejunum tissue slides (×100, upper panel) and (×400, lower panel). (**B**) HE staining of the colon in different groups (×100, upper panel) and (×400, lower panel). CON: control group without ovalbumin sensitization and challenge; OVA: allergic model group with ovalbumin sensitization and challenge; PSL: allergic model mice with prednisolone treatment; LRS: allergic model mice with lotus-seed resistant starch treatment. Arrow marks indicate the severe villi shedding, deepening of crypt depth, and intestinal tissue shedding.

Food allergy can lead to broad intestinal environmental changes. Small intestine (jejunum) and large intestine (colon) histologic analysis also supported the protective effects of lotus-seed resistant starch against food allergy. As shown in Figure 2, the gut villus atrophy and mucosa damage were more obvious in the allergic mice compared with non-allergic control mice, which were mitigated in the mice that received lotus-seed resistant starch.

Taken together, these results indicate that lotus-seed resistant starch could alleviate systemic allergic reactions in the OVA-induced food allergy model.

### 3.2. Lotus-Seed Resistant Starch Lowered Serum Concentrations of OVA-Specific Immunoglobulins and Allergy-Related Cytokine

**Figure 3:**
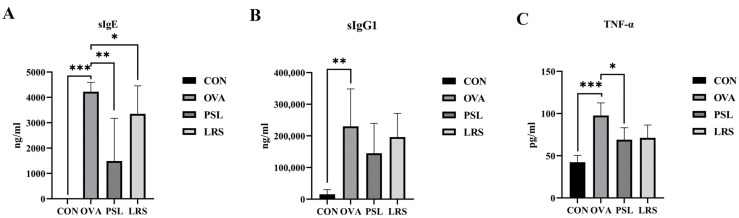
(**A**) The serum levels of OVA-specific IgE. (**B**) The serum levels of OVA-specific IgG1. (**C**) The serum levels of TNF-α. CON: control group without ovalbumin sensitization and challenge; OVA: allergic model group with ovalbumin sensitization and challenge; PSL: allergic model mice with prednisolone treatment; LRS: allergic model mice with lotus-seed resistant starch treatment. * *p* < 0.05, ** *p* < 0.01, *** *p* < 0.001 compared to OVA group.

Allergen-specific IgE and IgG1 are both important indicators of food allergy [14]. To further confirm the protective effects of lotus-seed resistant starch against OVA-induced allergy, the serum levels of allergen-specific IgE and IgG (two major mediators for triggering allergic response) were determined. As shown in Figure 3, the OVA-specific IgE and IgG1 content in the blood dramatically increased upon oral OVA administration, while the lotus-seed resistant starch significantly reduced IgE production in mice with an allergic state (Figure 3A). Meanwhile, the serum levels of IgG1 also showed a tendency to decrease upon lotus-seed resistant starch treatment, although statistical significance was not achieved (Figure 3B). Furthermore, we also determined the changes in TNF-α, a pro-inflammatory cytokine, reflecting the strength of the inflammatory response caused by food allergy [15]. Here, the serum levels of allergy-related cytokine TNF-α were also found to effectively decreased upon oral administration of lotus-seed resistant starch (Figure 3C).

### 3.3. Lotus-Seed Resistant Starch Intervention Regulated Th1/Th2 Balance and Lowered Serum Concentrations of Th2 Cytokines in OVA-Induced Allergic Mice

The splenic lymphocytes were isolated from all groups of mice after sacrifice, and the distribution of Th1 and Th2 cell populations was detected by flow cytometry. As shown in Table 1, compared to the control group, OVA immunization had a tendency to increase the proportion of Th1 (2.15 ± 1.34% vs. 1.36 ± 0.54%, *p* < 0.05), while Th2 proportion increased significantly (2.60 ± 0.78% vs. 1.17 ± 0.76%, *p* < 0.05), resulting in a Th1/Th2 imbalance towards Th2 due to the occurrence of food allergy with a difference inTh1/Th2 ratios between the control group and the OVA-induced group felling just short of statistical significance (*p* = 0.0655) (Table 1, Supplementary Figure S1). Meanwhile, both the prednisolone and lotus-seed resistant starch intervention slightly reduced the Th1 proportion and decreased the Th2 cell proportion to 2.08 ± 0.78% and 1.32 ± 0.80%, respectively, leading to overall increases in Th1/Th2 ratio (Table 1), suggesting that the food allergy associated Th2 cell differentiation might be downregulated by lotus-seed resistant starch.

**Figure 4:**
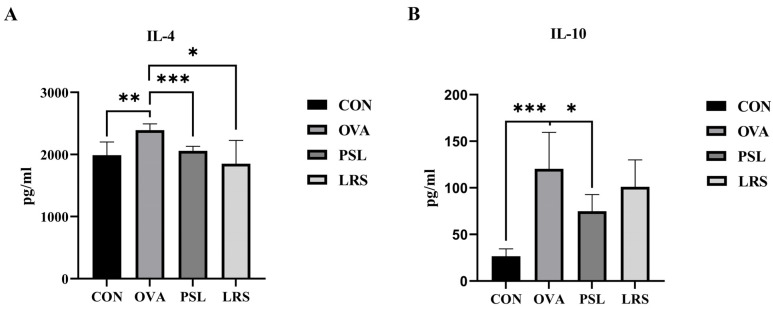
(**A**) The serum levels of IL-4. (**B**) The serum levels of IL-10. CON: control group without ovalbumin sensitization and challenge; OVA: allergic model group with ovalbumin sensitization and challenge; PSL: allergic model mice with prednisolone treatment; LRS: allergic model mice with lotus-seed resistant starch treatment. * *p* < 0.05, ** *p* < 0.01, *** *p* < 0.001 compared to OVA group.

In addition, Th1/Th2 imbalances are often associated with the dysregulation of Th2 cytokines, such as the dramatic elevation in IL-4 and IL-10. Consistent with the flow cytometry results, serum levels of Th2 cytokines (IL-4 and IL-10) were also found to be elevated in mice in the FA-induction group. Meanwhile, mice orally administered with lotus-seed resistant starch showed a significant tendency of suppression of IL-4 production (Figure 4A). Interestingly, although prednisolone resulted in a significant decrease in serum level of IL-10, lotus-seed resistant starch demonstrated little effect on IL-10 level (Figure 4B).

### 3.4. Lotus-Seed Resistant Starch Treatment Modulated the Gut Microbiota Composition, Fecal Short-Chain Fatty Acids, and Bile Acids of Mice with Food Allergies

**Figure 5:**
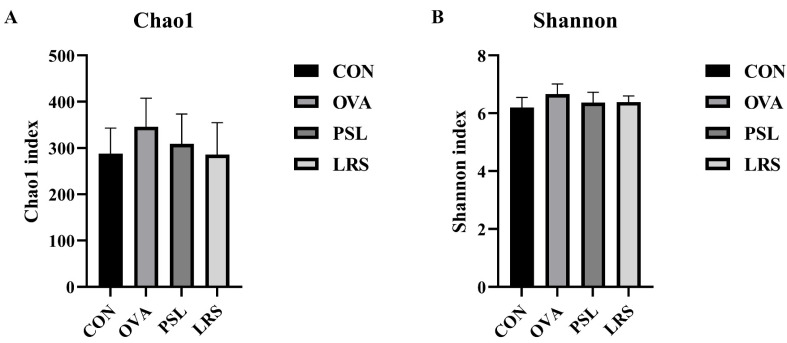
(**A**) The species richness estimate (Chao1) and (**B**) the diversity index (Shannon) of fecal samples from different groups. CON: control group without ovalbumin sensitization and challenge; OVA: allergic model group with ovalbumin sensitization and challenge; PSL: allergic model mice with prednisolone treatment; LRS: allergic model mice with lotus-seed resistant starch treatment.

**Figure 6:**
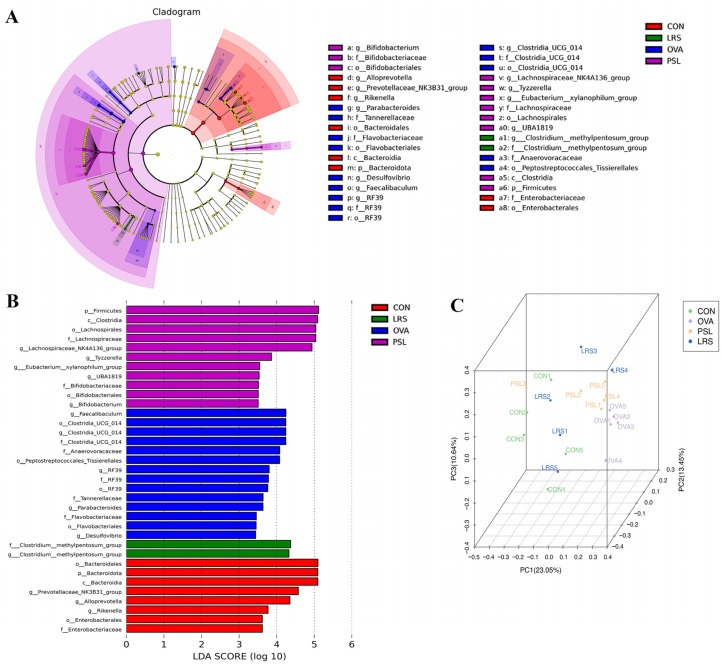
(**A**) Cladograms for the fecal microbial community generated from LEfSe analysis. Circles from the inside out represent taxonomic levels from phylum to genus. Each small circle at a different classification level represents a classification at that level, and the size of the small circle’s diameter is proportional to the size of the relative abundance. The species names represented in English are shown in the legend on the right. (**B**) LDA estimates the magnitude of the effect of the abundance of each species on the differential effect. The length of the bar chart represents the influence of significantly different species. The threshold of the logarithmic score of LDA analysis was 2.0. Differences between the control, model, prednisolone, and LRS groups. (**C**) Principal components analysis (PCoA) of 16S rRNA gene-sequencing analysis of fecal microbiota composition. CON: control group without ovalbumin sensitization and challenge; OVA: allergic model group with ovalbumin sensitization and challenge; PSL: allergic model mice with prednisolone treatment; LRS: allergic model mice with lotus-seed resistant starch treatment.

Next, the fecal microbiota composition was determined using 16S rDNA high-throughput sequencing to explore the effects of the lotus-seed resistant starch on the gut microbiota. As shown in Figure 5, the results revealed that the richness and diversity of the microbiota among groups were similar. However, as revealed by Linear discriminant analysis Effect Size (LEfSe) analysis, significantly different compositions in the gut microbiome were identified. The obvious differences in bacterial abundance at various taxonomic levels were observed (Figure 6A). The LDA effect size was shown by the histogram. In total, 48 differentially enriched bacterial taxa were discovered among groups, with 5 differentially abundant taxa at the phylum level and 17 differentially abundant taxa at the genus level (Figure 6B). The PCoA analysis also revealed that the fecal microbiota composition of allergic mice was distinct from that of control mice, while lotus-seed resistant starch treatment also resulted in obvious changes in the fecal microbiota composition (Figure 6C).

**Figure 7:**
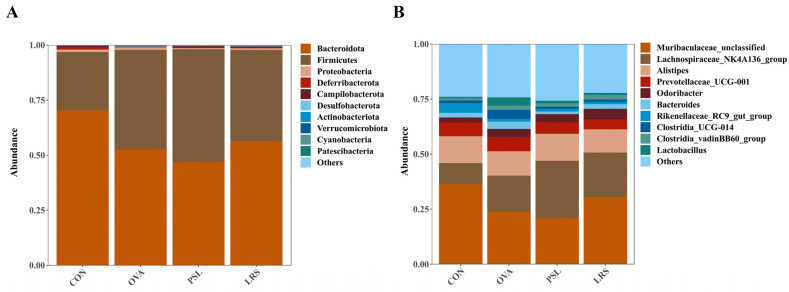
The differences in gut microbiota among groups at different levels. (**A**) Phylum level, (**B**) genus level. CON: control group without ovalbumin sensitization and challenge; OVA: allergic model group with ovalbumin sensitization and challenge; PSL: allergic model mice with prednisolone treatment; LRS: allergic model mice with lotus-seed resistant starch treatment.

To further understand the changes in the intestinal flora of food-allergic mice upon intervention, taxonomic compositions were further analyzed. At the phylum level (Figure 7A), the *Bacteroidetes* (70.40%) and *Firmicutes* (26.47%) were identified as dominant groups of gut microbiota in the control group. Interestingly, the *Bacteroidetes* abundance decreased to 52.44% in OVA-induced allergic mice, while lotus-seed resistant starch treatment increased the *Bacteroidetes* abundance to 56.35%.

Notably, OVA exposure slightly increased the relative abundance of *Proteobacteria* (1.10% to 1.15%) compared to normal mice, while prednisolone resulted in significant decreases in *Proteobacteria* (0.58%, *p* < 0.05).

At the genus level, compared with OVA mice, the relative abundance of probiotic bacteria such as *Muribaculaceae_unclassified* and *Alloprevotella* were found to be obviously higher in normal control mice (36.36 vs. 23.85%, and 3.96% vs. 0.17%, respectively), while lotus-seed resistant starch treatment nearly restored the abundance of *Muribaculaceae_unclassified* to 30.49% and effectively increased the *Alloprevotella* ratio to 1.10% significantly (*p* < 0.05). Interestingly, the level of proinflammatory bacteria *Clostridia_UCG-014* was significantly elevated upon OVA administration (1.11% to 4.11%, *p* < 0.01), and both prednisolone and lotus resistant starch treatment significantly reversed this increasing trend of *Clostridia_UCG-014* abundance (0.89%, *p* < 0.01 and 1.09%, *p* < 0.01, respectively).

Notably, the gut microbiota metabolites short-chain fatty acids and bile acids have been suggested to play pivotal roles in the regulation of food allergy. Here, the general decreases in the fecal short-chain fatty acids level were observed, while lotus-seed resistant starch partially recovered these changes, with the increases in isovalerate reaching statistical significance (Table 2). Similarly, the altered constitution of fecal bile acids was also found to be obvious in the OVA group, while, as shown in Table 3, the contents of bile acids (e.g., glycocholic acid (GCA) and glycolithocholic acid (GLCA)) were effectively restored after the intervention of lotus-seed resistant starch.

**Figure 8:**
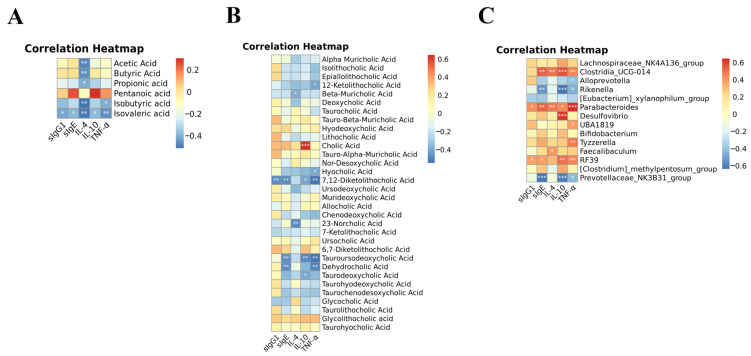
The correlation heatmaps represent significant statistical correlation values (r) between allergic responses and altered fecal short-chain fatty acid (**A**), altered fecal bile acids (**B**) and microbiota genera (**C**). * significant correlations with *p* < 0.05; ** *p* < 0.01, *** *p* < 0.001.

Indeed, the Pearson correlation analysis also suggested that microbial short-chain fatty acids production (especially isovaleric acid) was significantly negatively correlated with food allergic responses (Figure 8A). Indeed, a previous study also reported that food allergies often lead to intestinal inflammation, with low decreases in isovaleric acid being observed [16]. Similarly, a statistically significant correlation between bile acids and allergic reactions was also identified (Figure 8B). Furthermore, *Parabacteroides* (reported to be a strain of harmful bacteria [17]), *Clostridia_UCG-014* (a bacterium commonly associated with colon inflammation [18]), and *RF39* were found to be significantly positively correlated with a number of food allergy symptoms tested in our study, while *Rikenell* (previously identified to be a bacterium producing short-chain fatty acid [19]) and *Prevotellaceae_NK3B31_group* showed inverse correlations (Figure 8C). Furthermore, the relative abundance of *Alloprevotella* showed a negative correlation to serum TNF-α level (Figure 8C). Interestingly, a previous study suggested that *Alloprevotella* could be an OVA-induced allergy marker [20], which is also in accordance with our findings.

## 4. Discussion

Food allergy has drawn the attention of scholars over the past few decades due to the rapid increase in its prevalence. Indeed, a variety of food items can cause allergic reactions. with the most typical food allergens being peanut, tree nuts, cow’s milk, eggs, fish, wheat, soya, crustacean shellfish, etc. [21]. OVA is an antigen commonly used to establish animal models of food allergy, which features many typical allergic responses, such as the increase in antigen-specific IgE, intestinal inflammation, and changes in body temperature [22]. Here, these allergic reaction symptoms were also observed in the mice with OVA sensitization and challenge, indicating that the rodent model of food allergy was successfully and could be used to evaluate the potential prevention strategies for food allergy.

Lotus-seed resistant starch is a type 3 resistant starch formed by gelatinization and regeneration of lotus seed starch, which has been considered to be a dietary fiber because of its resistance to gastrointestinal digestion [23]. Indeed, the health beneficial effects of lotus-seed resistant starch (such as blood glucose and lipid-lowering effects) have gained increasing interest during the last decade, which might be attributed to its prebiotic activity (e.g., promoting the proliferation of *Bacteroidetes*) [10].

Notably, the vital roles of gut microbiota in modulating allergic reactions to certain foods have been gradually recognized in recent decades. Increasing evidence has highlighted the great potential of the interference of the gut microbiome as an effective way to curb the reaction of food allergy [24,25]. Therefore, gut-microbiota-targeted dietary intervention was considered a promising therapeutic strategy to manage and ameliorate food allergy [26]. For instance, the intake of high fiber, which is known to increase probiotics and decrease pathogenic bacteria in intestinal microflora, was proven to reduce the risk of food allergy or alleviate systemic allergic reactions [27]. Prebiotic supplementation was also reported to be associated with the alleviation of allergic reactions to food. Oral administration of *bifidobacterium* was reported to inhibit Th2 immune responses and improve impaired intestinal epithelial barrier function in food-allergic mice [28]. Here, the lotus-seed resistant starch was also found to exert anti-allergic activity in an OVA-induced rodent model. Furthermore, profound impacts on the gut microbiota composition have been observed in the allergic mice receiving lotus-seed resistant starch, which may be mechanisms underlying its allergy-alleviation activity. For instance, the lotus-seed resistant starch effectively augments the abundance of *Muribaculaceae_unclassified* and *Alloprevotella*, which were both found to be associated with increased short-chain fatty acids production in the gut [29,30]. In addition, the increase in *Alloprevotella* has also been proven to be beneficial for maintaining intestinal barrier integrity [30]. Furthermore, *Clostridia_UCG-014*, a pathogenic bacterium that exists in the inflammation of the colon [18], also increased drastically in allergic mice and appeared to decrease after LRS intervention. Admittedly, other possible underlying mechanisms could not be ruled out based on the results obtained in our study. For example, the alleviated pathological changes in the jejunum of mice with lotus-seed resistant starch treatment may result from the alleviation of jejunal inflammation. However, considering its indigestible property, it is reasonable to attribute the anti-allergic effects of louts seed starch, at least partially, to its regulatory activity on intestinal microbiota. In addition, the constitution of gut microbial metabolites (short-chain fatty acids (e.g., isovaleric acid) and bile acids (e.g., GCA and GLCA)) was found to be significantly altered upon lotus-seed resistant starch intervention in OVA-sensitized mice. Considering that previous studies already reported the association between isovaleric acid [31], GCA, GLCA [32], and food allergy, this observation also supports the notion that the impacts on gut microbiota may contribute, at least partially, to the anti-allergic activity of lotus-seed resistant starch.

Among immune cells, studies have already uncovered that T cells play essential roles in the development of food allergies. In particular, the Th1/Th2 imbalance towards Th2 and the dysregulation of Th2 cytokines can drive allergic responses by producing a number of cytokines, including IL-4, IL-5, and IL-10 [33]. For example, Hwang et al. reported that the percentages of Th2 cells were higher in OVA-sensitized mice than those in the control group. In their experiment, the proportion of Th2 cells was decreased, and the number of Th1 cells was increased by the administration of *Aster yomena,* with obvious anti-allergy effects being observed [34]. Studies from Duke University even directly altered the Th1/Th2 ratio in lymph nodes by using IL-12-containing particles to alleviate against the peanut antigen [35]. This strongly demonstrated that restoring the Th1/Th2 balance is an effective way to alleviate food allergy. Indeed, the latest findings suggest the crosstalk between the gut microbiome and Th1/Th2 balance. For example, *Lactobacillus rhamnosus* GG (LGG) was reported to induce immune tolerance against cow’s milk allergy by suppressing Th2 response [36]. Another in vivo study also revealed that *Bacillus coagulans (Bc), L. plantarum (Lp),* and *Bifidobacterium infantis* induce immunogenic tolerance against shrimp allergen tropomyosin by modulating Th2 level and balancing Th1/Th2 profile. Here, our obtained results also indicate that the beneficial effects of lotus-seed resistant starch may rely on its impact on Th1/Th2 balance, though future investigations are required to gain a better understanding of the possible underlying mechanisms.

## 5. Conclusions

In conclusion, our experimental results show that the intervention of lotus-seed resistant starch could alleviate the adverse symptoms in OVA-induced food-allergic mice. The underlying mechanisms might involve the regulatory effects on the gut microbiota, as well as the impact on Th1/Th2 balance. These findings may provide initial evidence to support the idea that dietary fibers may present promising candidates that could reduce food allergy by targeting the gut microbiota.

## Figures and Tables

**Table 1:** The proportion of Th1, Th2 cells in the spleen and the Th1/Th2 ratios.

CD4 + T cell content	CON	OVA	PSL	LRS
Th1 (%)	**1.36 ± 0.54** *	2.15 ± 1.34	2.07 ± 0.87	1.86 ± 0.88
Th2 (%)	**1.17 ± 0.76** *	2.60 ± 0.78	2.08 ± 0.78	**1.32 ± 0.80** *
Th1/Th2	1.39 ± 0.49	0.81 ± 0.36	1.10 ± 0.67	**1.49 ± 0.50** *

CON: control group without ovalbumin sensitization and challenge; OVA: allergic model group with ovalbumin sensitization and challenge; PSL: allergic model mice with prednisolone treatment; LRS: allergic model mice with lotus-seed resistant starch treatment. * *p* < 0.05 compared to the OVA group.

**Table 2:** The fecal short-chain fatty acids profiles.

SCFAs (μg/g)	Control	OVA	PSL	LRS
Acetic acid	**1194.48 ± 124.82 ^a^**	1037.60 ± 195.36 ^b^	1154.31 ± 250.92 ^b^	1205.22 ± 302.80 ^b^
Butyric acid	**394.66 ± 97.40 ^a^**	306.99 ± 95.27 ^b^	373.35 ± 161.86 ^b^	436.92 ± 139.79 ^b^
Isobutyric acid	**22.80 ± 2.24 ^a^**	13.64 ± 3.75 ^b^	19.68 ± 8.33 ^b^	18.97 ± 7.18 ^b^
Isovaleric acid	**14.47 ± 0.94 ^a^**	8.10 ± 1.31 ^b^	**12.80 ± 5.59 ^a^**	**11.36 ± 4.13 ^a^**
Pentanoic acid	17.65 ± 3.64	20.46 ± 4.42	21.00 ± 8.86	19.06 ± 8.19
Propionic acid	**364.59 ± 53.03 ^a^**	274.69 ± 65.62 ^b^	280.89 ± 79.36 ^b^	304.34 ± 116.38 ^b^

CON: control group without ovalbumin sensitization and challenge; OVA: allergic model group with ovalbumin sensitization and challenge; PSL: allergic model mice with prednisolone treatment; LRS: allergic model mice with lotus-seed resistant starch treatment. Different letters indicate statistically significant differences (*p* < 0.05).

**Table 3:** The fecal bile acid profiles.

Bile Acids (ng/g)	CON	OVA	PSL	LRS
CDCA	149.63 ± 82.16	77.87 ± 86.60	117.74 ± 95.83	89.88 ± 67.16
CA	732.98 ± 344.10	1041.95 ± 390.68	831.78 ± 167.68 *	939.84 ± 465.74
GCA	12.92 ± 6.83	10.20 ± 6.33	10.15 ± 9.07	3.93 ± 3.02 *
TCDCA	74.95 ± 89.24	42.22 ± 25.71	40.56 ± 21.48	31.97 ± 22.89
TCA	2194.48 ± 1419.93	1867.41 ± 927.63	1836.84 ± 674.11	1687.60 ± 379.88
LCA	1020.97 ± 581.76	733.73 ± 939.00	1325.27 ± 1042.11	884.69 ± 594.49
DCA	2312.20 ± 1845.32	1940.72 ± 663.47	3702.57 ± 1486.00	2704.53 ± 1942.32
UDCA	168.62 ± 104.83	121.00 ± 98.37	233.62 ± 89.91 *	170.46 ± 106.78
GLCA	1.34 ± 1.72 *	7.12 ± 5.53	6.07 ± 4.00	2.81 ± 1.99 *
TLCA	8.98 ± 11.71	6.46 ± 2.51	7.48 ± 3.06	4.34 ± 3.48
TUDCA	139.41 ± 105.88	23.54 ± 17.04	92.73 ± 70.15	60.68 ± 50.33
TDCA	98.91 ± 100.31	31.99 ± 21.70	81.34 ± 40.87	56.67 ± 72.48
EALCA	6187.93 ± 2638.41	3736.37 ± 4052.82	5291.85 ± 4361.71	4948.35 ± 1971.00
HDCA	1133.45 ± 702.83	1390.67 ± 674.78	1404.02 ± 689.62	1281.71 ± 810.33
α-MCA	16,125.42 ± 5693.38	12,831.89 ± 3895.25	19,127.22 ± 7009.88	14,603.84 ± 5598.79
β-MCA	4397.20 ± 3288.10	3293.72 ± 919.33	5699.42 ± 1437.25 *	4071.21 ± 1424.47
α-TMCA	324.92 ± 192.09	387.32 ± 51.84	530.31 ± 165.58	378.89 ± 122.55
THDCA	74.95 ± 89.24	42.22 ± 25.71	40.56 ± 21.48	31.97 ± 22.89
Iso-LCA	6958.30 ± 3205.64	3851.44 ± 4773.45	5905.51 ± 5359.43	5452.63 ± 2410.86
M-DCA	137.60 ± 129.33	158.93 ± 86.15	213.68 ± 87.20	152.94 ± 110.68
β-TMCA-Na	1146.03 ± 734.61	1351.69 ± 258.84	1882.41 ± 683.18	1296.47 ± 452.58

CON: control group without ovalbumin sensitization and challenge; OVA: allergic model group with ovalbumin sensitization and challenge; PSL: allergic model mice with prednisolone treatment; LRS: allergic model mice with lotus-seed resistant starch treatment. * *p* < 0.05 compared to the OVA group.

## Data Availability

Data are contained within the article. The 16s ribosomal sequencing data can be accessed via the link: https://www.ncbi.nlm.nih.gov/bioproject/PRJNA912321 (accessed on 1 February 2023).

## Supplementary Materials

The following supporting information can be downloaded at: https://www.mdpi.com/article/10.3390/foods12040737/s1, Figure S1: Representative flow cytometry dot plots depicting Th1 and Th2 staining; Table S1: Primers specific for 16S rDNA.
