# Supplementary material for: Oral Administration of Lotus-Seed Resistant Starch Protects against Food Allergy
Source: Foods. 2023 Feb 8;12(4):737. doi: 10.3390/foods12040737 (PMC9956242; doi:10.3390/foods12040737)
Supplement: Supplementary file 1 [file foods-12-00737-s001.zip › foods-2143047-SI.pdf]

Supplementary Table S1

Table S1. Primers specific for 16S rDNA.

Primer	Sequence (5'-3')
Forward	TACGGRAGGCAGCAG
Reverse	AGGGTATCTAATCCT

Supplementary Figure S1

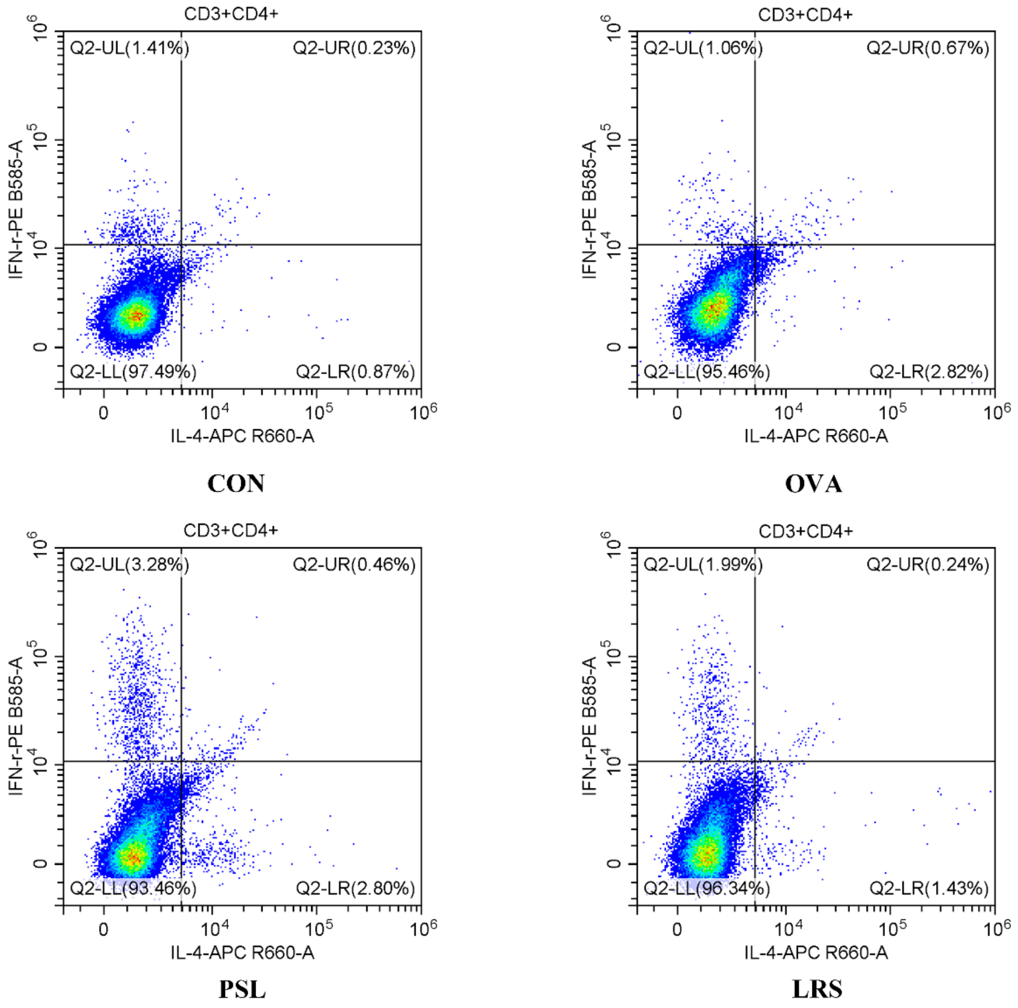

Figure S1. Representative flow cytometry dot plots depicting Th1 and Th2 staining.
